# Building a Culture of Health at the Neighborhood Level Through Governance Councils

**DOI:** 10.1007/s10900-020-00804-0

**Published:** 2020-03-13

**Authors:** Jennifer Pierre, Carl Letamendi, Luke Sleiter, Zinzi Bailey, Rachel Dannefer, Lauren Shiman, Jaime Gutierrez, Elana Martins, Richard Sierra

**Affiliations:** 1 Bureau of Brooklyn Neighborhood Health, Center for Health Equity and Community Wellness, New York City Department of Health and Mental Hygiene, 485 Throop Avenue, 2nd Floor, Room 2467, Brooklyn, NY 11221 USA; 2 Bureau of Equitable Health Systems, Center for Health Equity and Community Wellness, New York City Department of Health and Mental Hygiene, Queens, USA; 3 Present Address: Ology Research Group, New York, USA; 4 Bureau of Alcohol and Drug Use Prevention, Care and Treatment, Division of Mental Hygiene, New York City Department of Health and Mental Hygiene, Queens, USA; 5 Health Equity Research Solutions, LLC, Miami, FL USA; 6 Bureau of Harlem Neighborhood Health, Center for Health Equity and Community Wellness, New York City Department of Health and Mental Hygiene, New York, USA; 7 Bureau of Bronx Neighborhood Health, Center for Health Equity and Community Wellness, New York City Department of Health and Mental Hygiene, Bronx, USA; 8 Bureau of Brooklyn Neighborhood Health, Center for Health Equity and Community Wellness, New York City Department of Health and Mental Hygiene, Brooklyn, USA

**Keywords:** Cross-sector collaboration, Neighborhood health, Health equity, Place-based interventions, Local health department

## Abstract

To explore facilitators and barriers to developing and sustaining collaboration among New York City Department of Health and Mental Hygiene’s Neighborhood Health Action Centers and co-located partners, who share information and decision-making through a Governance Council structure of representative members. Semi-structured interviews were conducted in 2018 with 43 Governance Council members across the three Action Centers of East Harlem (13), Tremont (15), and Brownsville (15), New York City. Governance Council members identified collaboration through information- and resource-sharing, consistent meetings and continuous communication as valuable for fostering a culture of health in their communities. Immediate benefits included building relationships, increased access to resources, and increased reach and access to community members. Challenges included difficulty building community trust, insufficient advertisement of services, and navigation of government bureaucracy. The Governance Councils forged collaborative relationships among local government, community-based organizations and clinical providers to improve health and well-being in their neighborhoods. Sharing space, resources and information is feasible with a movement towards shared leadership and decision-making. This may result in community-driven and tailored solutions to historical inequities. In shared leadership models, some internal reform by Government partners may be required.

## Introduction

Concurrent with growing recognition that social and structural factors heavily impact health and well-being [1, 2], variables such as geographic location and social environment have been identified as crucial determinants of personal and population health [3]. For New York City residents living in neighborhoods like East Harlem, Tremont and Brownsville, historical injustices, racist practices and policies have worsened environmental conditions and perpetuated poor health outcomes [4–6]. These neighborhoods have the highest rates of premature mortality and chronic disease in New York City, with cancer, heart disease, HIV, and drug-related conditions being among the leading causes of premature mortality (Table 1) [7–9].

**Table 1:** Key population characteristics, by neighborhood health action center community district

	East Harlem (Manhattan CD 11)	East Tremont (Bronx CD 6)	Brownsville (Brooklyn CD 16)
Population size	124,323	87,476	84,525
Race/ethnicity			
Latino	50%	67%	20%
Black	30%	25%	76%
White	12%	6%	1%
Asian	6%	1%	1%
Other	2%	1%	2%
Foreign-born	24%	31%	30%
Limited english proficiency	19%	27%	10%
Poverty	23%	31%	28%
Unemployment	11%	16%	14%
Causes of premature death, by rank			
1	Cancer	Heart disease	Cancer
2	Heart disease	Cancer	Heart disease
3	HIV	Drug-related	HIV
4	Drug-related	HIV	Homicide
5	Accidents	Diabetes mellitus	Drug-related

**Fig. 1:**
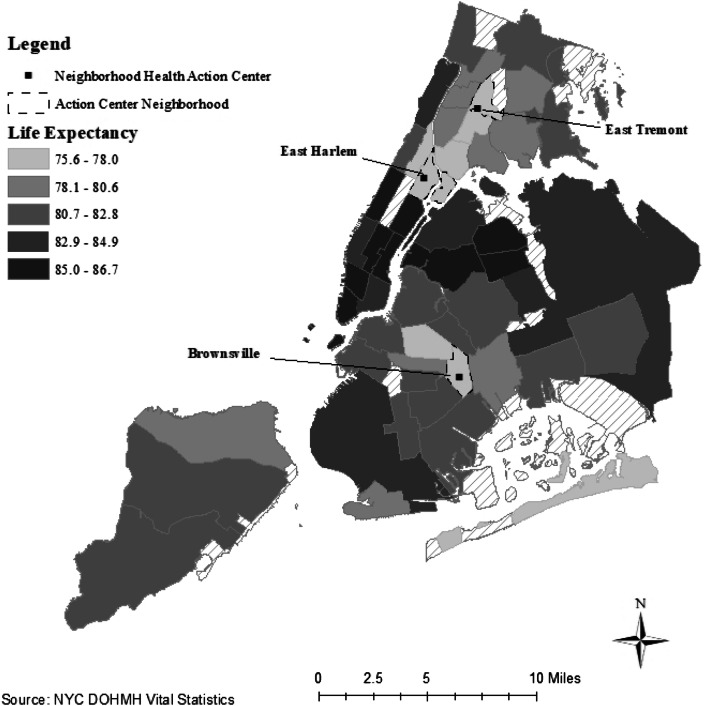
Life expectancy at birth by community district of residence, New York City, 2008–2017

The New York City Department of Health and Mental Hygiene (Health Department) takes an intentional approach to improving community health through a place-based model which addresses health determinants and their root causes. One such approach was the development of Neighborhood Health Action Centers (Action Center) as part of a neighborhood health strategy to invest in historically disinvested neighborhoods which bear disproportionate burdens of premature mortality. This strategy is comprised of three components that address social and institutional issues which affect health: co-location of services and a robust referral system; innovation in programs and policies, which incorporates data and resident feedback to shape programs, systems and policies; and community engagement, action and impact [10]. The first three Action Centers included East Harlem Action Center in Manhattan Community District 11, Tremont Action Center in Bronx Community District 6, and Brownsville Action Center in Brooklyn Community District 16 (Fig. 1) [11].

As part of operationalizing the first component, Action Centers co-located Health Department, clinical, and community-based service providers under one roof in a place-based model that facilitates collaboration (Table 2). This began with the Health Department releasing a Request for Expression of Interest in 2015 for non-profit organizations or government entities who were willing to contribute to its mission to improve health for residents with an equity approach. Through a license agreement, these entities would occupy space across the agency’s underutilized public buildings to provide health-related services to underserved New Yorkers. The vision was to create a place where neighborhood residents could receive services and participate in health promoting activities at low or no cost. Eligible partners were those who provided primary care health services, dental services, community health worker programs, social promotion and violence prevention, family support services, healthy eating and food services, active living and built environment programs, home visiting for maternal health, behavioral and mental health services, health insurance navigation and enrollment, and youth health services.

**Table 2:** Governance council members by neighborhood health action center

Neighborhood	Partners	Organizations and programs	Sector
Tremont	Co-located partners	Health and Hospital Gotham Health	Healthcare Social service
		NYC Smoke Free (Public Health Solutions)	
	Co-located Health Department programs	Bureau of HIV	City Government
		Bureau of Bronx Neighborhood Health	
		Bureau of Operations	
		Behavioral Health	
		Office of School Health	
		Pest Control	
	External Health Department Partners/Programs	Bronx Diabetes Prevention Partnership	City Government
		Early Intervention	
		Newborn Home Visiting	
		Bureau of STI	
		Office of Faith-Based Initiatives	
		Shop Healthy Bronx	
		Condom Distribution	
Brownsville	Co-located partners	Brownsville Multiservice Family Health Center	Healthcare/Social Service Healthcare Social service/CBO
		Health and Hospital Gotham Health	
		Brooklyn Perinatal Network	
	Co-located Health Department programs	Bureau of Brooklyn Neighborhood Health	City Government
		Healthy Start Brooklyn	
		Family Wellness Suite	
		Behavioral Health	
		Shop Healthy	
		Friendship Benches	
		Office of School Health	
		Bureau of Operations	
	External Partners	Catholic Charities Maimonides Medical Center	Social service/CBO Healthcare
East Harlem	Co-located partners	Association to Benefit Children	Social service/CBO
		Concrete Safaris	Social service/CBO
		ID NYC	Local gov’t (other)
		Public Health Solutions	Social service/CBO
		SMART University	Social service/CBO
	Co-located Health Department programs	Bureau of Harlem Neighborhood Health	City Government
		Family Wellness Suite	
		Harlem Health Advocacy Partnership	
		Bureau of Operations	
		Newborn Home Visiting	
		Pest Control	

Applicants awarded space in the building were contractually obligated to participate in neighborhood health planning efforts—working collectively with an array of neighborhood stakeholders and service providers to leverage assets, develop shared goals, and coordinate actions to achieve systems change that addresses the unique needs of the community served. They were also obligated to participate in the Action Center’s Governance Council, a collective decision-making body, made up of representatives from respective co-located entities, by providing consistent and stable representation at meetings.

The Councils meet monthly to plan, coordinate and resolve building operations issues, develop collaborative activities and implement programs that serve local community needs. Meetings are convened, coordinated and facilitated by the Health Department. These meetings began a few months after the Action Centers opened, which occurred in a rolling nature between September 2016 and April 2017.

The format of the monthly Governance Council meetings across Action Centers is similar. Meetings are convened, coordinated and facilitated by the Health Department. At each meeting, representative members introduce their programs and share updates, information and announcements. Pertinent operation issues or joint endeavors are discussed, and co-located partners or invited external partners share details about their programs and services.

While the structure of East Harlem and Tremont’s Governance Councils are similar—one Council meeting comprised of representatives from each program and co-located partners—Brownsville’s is made up of two arms, an Executive Governance Council which is comprised of members with decision-making authority assigned by their respective organizations, and a General Governance Council which is comprised of members of the Executive Governance Council, other staff and external partners. Issues that need resolving are discussed at the Executive Governance Council meeting, which typically follows the General Governance Council meeting.

An earlier evaluation of the Harlem Action Center focused on the early implementation outcomes of the first Action Center established, and gathered information from visitor surveys, monitoring of referral data, and qualitative interviews with stakeholders [10]. This current study expands on that evaluation with the specific intent to consider how the exchange of information, ideas and the formation of collaborative partnerships supported by the Governance Councils can contribute to a culture of health at the neighborhood level, as laid out in the RWJF’s Systems for Action framework, specifically Area 2: Fostering Cross-Sector Collaboration to Improve Well-Being [12–14].

This paper shares findings from semi-structured interviews with Governance Council members from all three Action Centers that explored facilitators and barriers in developing and sustaining collaboration among the Action Centers’ co-located partners.

## Methods

### Governance Council Interviews

During 2018, semi-structured interviews were conducted with Governance Council members across all three Action Center sites. This allowed us to compare experiences across Action Centers, especially as the model was being adapted to different neighborhoods and partnerships. Members were eligible to be interviewed if they had attended at least three Governance Council meetings, and efforts were made to include a cross-section of co-located partners to gain various perspectives. Members were informed of the planned evaluation interviews by one of the three co-investigators of the study at a Governance Council meeting. Each Action Center provided a list of eligible members, and email invitations were issued by a research assistant, who followed up with a telephone call. Participation was voluntary. All interviews were conducted by the co-investigators who alternated the roles of interviewing and note-taking.

Before each interview, verbal consent to participate in the interview and to have the interview audio-recorded was obtained. A consent statement was read to each participant, explaining why they were invited to take part in the study, the aims of the interview, confidentiality measures, the voluntary nature of the interviews and how findings would be shared. Participants were also advised that they could skip any questions they did not wish to answer, and that they could discontinue the interview at any time. Interviews began after participants’ verbal consent to participate and to record the interviews. Participants were asked to answer questions about the Governance Council’s purpose and the impact that being in the Action Center has had on their organizations and programs. They were also asked to share their views about successes, challenges and recommendations for the Governance Council as well as the Action Center.

In East Harlem, 13 Governance Council members were interviewed between June and July 2018. In Tremont, 15 Governance Council members were interviewed in August 2018, and in Brownsville, 15 Governance Council members were interviewed in November 2018, 4 of whom were members of their Executive Governance Council.

Transcripts were prepared verbatim by the research assistant, re-checked for accuracy by one of the three co-Investigators, all of whom analyzed the data through a process of focused coding. The questions asked were used as broad domains, then each line of data was analyzed for themes and subthemes [15]. Common themes across all three sites are presented below. This study was approved as exempt research by the Health Department’s Institutional Review Board.

## Results

### Governance Council Purpose and Structure

#### Governance Council Provided an Anchor for Organic Interactions

Across sites, most Governance Council members stated that the Governance Council fosters collaboration among its co-located partners, brings them together to build cohesiveness, develop partnerships and work together as one body. Being at the Action Centers offered increased opportunities for cross-partner collaboration and relationship building, not only with co-located partners but with other community partners who they meet when they are invited to present about their programs. One East Harlem member referred to the dynamics that occur in the Governance Council as an “organic” process, where collaboration became a natural and continuous process as partners got to know each other and their respective work. Forming synergistic and beneficial relationships with other partners improved cohesion among co-located partners, giving members a sense of being part of a larger team.

We can collaborate with them around criminal justice and different things that, you know, people that we've spoken to really want to be engaged with. So, it's just great. It's like having a larger team, right within this building. (East Harlem Governance Council Member)

#### Governance Council Meeting Structure Encouraged Collaboration Through Consolidated Information and Resource Sharing

Many Governance Council members viewed the meetings as a platform for various organizations to come together, share information about their programs and learn about other programs. Besides program updates, members and external community organizations were invited to make presentations about their programs. Members particularly appreciated the structure of having guest speakers, as this gives them the opportunity to hear about other programs and services available in the community.

Information shared between partners at the meetings created opportunities to connect and have follow-up conversations, and in several cases, has resulted in increased access to resources for their clients, and increased referrals and connections among internal and external partners.

[The] Governance Council for me is a way for all of the programs to get together and talk about their services, and … the services that their outside partners provide. And it's really about information sharing, because that's really important. I feel like, a lot of times programs don't share information and we're kind of blind to what each other does. (Tremont Governance Council Member)

#### Governance Council Meeting Consistency Provided a Mechanism for Continuous and Effective Communication

Members reported feeling a sense of commitment fostered largely by meeting consistency. They felt that convening on a monthly basis has helped establish the meeting culture and sent the message that the Governance Council is a vital part of Action Center operations. Some members expressed that there were good processes for communication among partners that help facilitate planning for activities such as sharing meeting minutes and creating a monthly directory of events based on information shared by partners during the meeting.

I think like actually maintaining the meeting ... the tone is set that, you know these are important and this is part of what we do, and this is how we function. So, I think that that's an accomplishment in itself, because that's hard to do. And that takes leadership, you know. (East Harlem Governance Council Member).

Members reported that emergent issues such as building- or operations-related issues or staff trainings after a traumatic community event are addressed as they arise. They said the Governance Council meeting was a place where members felt free to voice their concerns and suggestions, which are considered and resolved at the meeting or elevated to leadership, as needed.

I think the once a month meeting really fosters that camaraderie to feel comfortable with everybody who's here … it makes you feel like you belong. (East Harlem Governance Council Member)

### Benefits

#### Relationship Building Within and Outside of the Action Center

Members shared that one of the benefits of co-location and the Governance Council structure was the facilitation of relationship building among members. They stated that there is now more “inter-connectedness” between partners in the building and this has resulted in an increased sense of having a shared identity as the Action Center. Members shared that the increased spirit of collaboration with other partner organizations allows them to leverage each other’s strengths to benefit their clients and the wider community. One member noted that new people representing external organizations are frequently invited to present at the meetings, which shows that new connections (and potentially access to additional resources) are continually being made.

I am glad to say they [the Governance Council] brought a sense of unity to this building. That was part of the problem in the beginning, there was no unity. We've got a sense of unity, we understand what each... clinic, what everybody is doing. (Brownsville Governance Council Member)

Members also reported that being at the Action Center has allowed them to build relationships with community residents. Because the Action Center is an open space for community residents, they reported that many have embraced the programs and are coming in repeatedly to utilize the resources. As a result, members are getting to know entire families and are beginning to better understand the needs of the community. One member shared that being at the Action Center has sensitized them to the depths of the inequities experienced by the community, and this has made them more aware, open, and willing to listen and learn to find ways to disseminate information about the resources available at the Action Center.

Well, I see a lot of regulars, like families, like the parents and the kids [and] we form relationships with them, with the babies … and they come to the program. So, I see a lot of interaction like that. A lot of the mothers, they come to a lot of programs and they're very involved. (Brownsville Governance Council Member)

#### Increased Access, Reach, and Cross Promotion

Members shared that being part of the Action Center has positively impacted their access and reach to the communities they serve. They reported increased access to community resources for clients through knowledge of Action Center resources or by learning about resources in the wider community that they could refer clients to. Many programs expanded with increasing referrals to their programs, and many have been able to reach and retain more clients.

Being at the Action Center has really given us access to a community that we previously didn't have access to. (East Harlem Governance Council Member)

Members also stated that they have benefited from increased access to staff support for program promotion and referral services at the Action Center. For example, partnering with Action Center health promoters to conduct outreach and program promotion have resulted in an increase in Action Center visits. Access to Action Center navigators who inform and direct clients to program services, health promoters who work to inform neighborhood residents about local health and social services, and referral specialists who assist with referrals both within and outside the Action Center has helped in communicating with clients and facilitating referrals to services. For example, uninsured visitors to clinical services are referred to a co-located partner who can help them navigate the Health Insurance Marketplace to enroll in health insurance.

When they have patients that come in and don't have health insurance or their health insurance has been discontinued, they'll refer people to [co-located community-based organization]. So I guess that's a success. You know, making sure that people have health insurance and can essentially receive the services. (Brownsville Governance Council Member)

Members also reported that they have found ways to share resources by engaging in the planning process together. Learning that many members attend the same events, they decided that they could work smarter and increase their capacity and attendance at events by cross-promoting programs.

### Challenges

#### Building Community Trust

While members acknowledged that there has been increased foot traffic through the Action Centers, they perceive that there is still a need to engage in activities that would build community trust. Some communities generally distrust government and may view the Action Center building as strictly a government entity. Related to this, each Action Center, like most government buildings, has a uniformed Health Department police officer, who is usually the first person a client sees when they enter the building. Members perceive that they may be intimidating to the public and may deter some neighborhood residents from visiting.

You know, I think there are still a lot of people out there that are difficult to engage … the Health Department has already made it very, very clear that, you know, coming to get services from the Health Department will not put them in danger. But still, there are segments of the population that are very untrusting and that has to do with like historical experiences. (Tremont Governance Council Member)

#### Insufficient Advertisement of Programs and Services

Members reported that there is still a lack of awareness of what the Action Centers offer, which is in part due to the lack of exterior signage advertising the services available in the building. This is especially important after hours when the building is closed. Members also stated that while they can and do advertise on the general Health Department social media platform, they feel they can be more impactful and visible if each Action Center were to have their own social media platform which is currently not the case.

Not having social media really makes their life (health promoters), their jobs harder and it makes us less visible. There's a bunch of flyers out there and that generic brand is just like not noticeable. (East Harlem Governance Council Member)

Additionally, one member pointed out that the Action Centers still grapple with a branding issue of being associated with services previously offered there, such as STD or HIV testing and treatment or immunizations. As such, they felt that community outreach is even more crucial to build awareness of the Action Center and its new programs and services, to garner community support and involvement.

I think inherently what the Action Centers have had and what they grapple with is a branding issue. Anecdotally, a lot of the residents that I’ve spoken to in passing when I told them where we sit, they say, “Oh, the AIDS clinic, the STD clinic.” (Brownsville Governance Council Member)

#### Navigating Government Bureaucracy

Members mentioned challenges in having to go through many layers of approval inherent in a government bureaucracy which sometimes create roadblocks for certain projects at the Action Centers. They stated that adherence to bureaucratic protocols can sometimes prohibit or hamper decision-making at the local level, and sometimes delay or prevent projects from moving forward. They indicated that this is a major obstacle when working with partners, especially when they cannot deliver on partners’ requests or promised services in a certain timeframe.

Some of the more ambitious projects are a little bit hard to get off the ground. They're good but, as soon as when you get to a certain spot, there are hard stops, not from us. It’s the politics or the red tape. (Brownsville Governance Council Member)

## Discussion

The RWJF Culture of Health Framework, Action Area 2, which seeks to foster cross-sector collaboration to improve the community’s well-being, identifies three main drivers for effective and sustainable cross-sector collaborations: (1) the number, breadth, and quality of partnerships, (2) the adequacy of investment in these partnerships, and (3) the adoption of policies to support them [13, 14].

The findings from the Governance Council interviews indicate significant progress on the first driver, yet poses questions about the remaining two. The three Governance Councils who share some decision-making responsibilities in the Action Centers have forged organic forms of collaboration in their neighborhoods, consistent with this framework. Even though the three Action Centers and their respective Governance Councils operated independently in different neighborhoods (Brownsville, Tremont and East Harlem), members across sites concurred that Governance Councils foster collaboration among co-located occupants of the Action Centers through sharing of information and resources, consistent meetings, and engaging in continuous communication. Because community-based organizations often want to connect with other organizations or health departments but the integration and coordination infrastructure is sometimes lacking [16, 17], the Governance Council model, as evidenced by our findings, can provide a feasible structure to facilitate improved communication and integration across partners from different sectors seeking to improve neighborhood health. In fostering information sharing and communication, this model helps to build relationships among Action Center staff, co-located partners and external partners that allow them to have increased reach and access to community members as well as access to additional resources for their clients.

Challenges in building community trust, advertising Action Center programs and services more widely, and navigating through government bureaucracy indicate that current investments in staff, capital improvements, communication infrastructure and structural support need to be shored up in order to maximize the impact and sustainability of the Governance Councils.

While there is still a lot more work to be done, some essential first steps have already been achieved. Achieving co-location of services under one roof is one such step in building cross-sector collaboration. Bringing the sectors together to share information and communicate with each other is another essential ingredient which has been successfully achieved by the Governance Council.

Collaborative work between local government and community organizations is possible, and there can be movement towards shared leadership and decision-making. The Governance Council Model can easily be replicated in other settings. However, government partners have the additional responsibility of internal reform to be responsive to shared leadership models. We believe that this will lead to more community-driven and tailored solutions to addressing historical inequities which have perpetuated the health outcomes we see in the neighborhoods we serve.
